# Factors associated with access and adherence to artemisinin‐based combination therapy (ACT) for children under five: a secondary analysis of a national survey in Sierra Leone

**DOI:** 10.1186/s12936-021-03590-9

**Published:** 2021-01-21

**Authors:** Kristin Banek, Emily L. Webb, Emily Bostick Doogue, Samuel Juana Smith, Daniel Chandramohan, Sarah G. Staedke

**Affiliations:** 1 Institute for Global Health and Infectious Diseases, University of North Carolina, Chapel Hill, USA; 2 Department of Clinical Research, London School of Hygiene and Tropical Medicine, Keppel Street, London, WC1E 7HT UK; 3 MRC Tropical Epidemiology Group, London School of Hygiene and Tropical Medicine, Keppel Street, London, WC1E 7HT UK; 4 Catholic Relief Services, Catholic Relief Services, 228 W. Lexington Street, Baltimore, MD 21201 USA; 5 National Malaria Control Programme, Freetown, Sierra Leone; 6 Department of Disease Control, London School of Hygiene and Tropical Medicine, Keppel Street, London, WC1E 7HT UK

**Keywords:** Malaria, Antimalarial, Artemisinin-based combination therapy (ACT), Sierra Leone, Prompt treatment, Access, Adherence, Treatment completion

## Abstract

**Background:**

Access and adherence to artemisinin-based combination therapy (ACT) are key challenges to effective malaria treatment. A secondary analysis of the Sierra Leone malaria Knowledge, Attitudes, and Practices (mKAP) survey was conducted to investigate access and adherence to ACT for the treatment of fever in children under-five.

**Methods:**

The mKAP was a nationally representative, two-stage cluster-sample survey, conducted in 2012. Thirty primary sampling units per district were randomly selected using probability proportionate to size, based on national census estimates; 14 households were subsequently randomly selected and enrolled per sampling unit. The analysis was restricted to children under-five with fever in the past two weeks. Factors associated with access and adherence were assessed using multivariate logistic regression.

**Results:**

Of 5169 enrolled households, 1456 reported at least one child under-five with fever in the past two weeks. Of the 1641 children from these households, 982 (59.8%) received any treatment for fever and were analysed for access to ACT; 469 (47.6%) received ACT and 466 were analysed for treatment adherence. Only 222 (47.4%) febrile children received ACT and completed 3-day treatment. In an adjusted analysis, factors associated with ACT access included knowledge of ACT (odds ratio [OR] 2.78, 95% CI 2.02–3.80; p < 0.001), knowledge of insecticide-treated nets (ITNs) (OR 1.84, 95% CI 1.29–2.63; p = 0.001), source of care (public health facility vs. other; OR 1.86, 95% CI 1.27–2.72, p = 0.001), geographic region (East vs. West; OR 2.30, 95% CI 1.20–4.44; p = 0.025), and age (24–59 vs. 0–23 months; OR 1.45, 95% CI 1.07–1.96; p = 0.016). The only factor associated with ACT adherence was time to treatment; children treated within 24 h were less likely to adhere (OR 0.55, 95% CI 0.34–0.89; p = 0.015).

**Conclusions:**

In 2012, access and adherence to ACT remained low in Sierra Leone. Knowledge of ACT and ITNs, and seeking care in the public sector, were most strongly associated with ACT access. National surveys provide important information on anti-malarial access and could be expanded to measure treatment adherence.

## Background

Malaria remains a serious health problem in sub-Saharan Africa and is particularly dangerous for children under-five [1–3]. Prompt access to effective treatment is critical to malaria control. The World Health Organization (WHO) malaria treatment guidelines recommend that all laboratory-confirmed malaria cases be treated promptly (within 24 h of onset) using artemisinin-based combination therapy (ACT) [4]. Despite this recommendation, access to prompt and effective treatment remains sub-optimal (on average, 65%) across sub-Saharan Africa [3]; with access to ACT influenced by availability, affordability, and acceptability [5]. Weak health systems are adversely impacted during global public health emergencies, such as Ebola and COVID-19, leading to disruptions in service provision, supply chain, and health-seeking behaviour [6, 7]. During the Ebola outbreak 2014–2015 in Sierra Leone, pre-existing gaps in reporting and service delivery worsened [8], and changes to service delivery had a lasting impact on access in neighbouring Liberia [9]. Although access to ACT is essential, multiple factors influence the effectiveness of treatment, including the efficacy of ACT regimens, targeted testing and treatment, and patient (or caregiver) adherence to treatment [10, 11].

In Sierra Leone, malaria is the leading cause of morbidity and mortality in children under-five, accounting for 47% of outpatient visits [12]. Although amodiaquine plus artesunate (AQ + AS) was recommended as the first-line treatment for uncomplicated malaria in 2004 [13], access to ACT remained low, with only 19.2% of children with fever receiving ACT in 2010 [14]. Furthermore, probable adherence to co-packaged AQ + AS in Sierra Leone was reported to be only 48.7% in 2008 [15]. In the 2016 Malaria Indicator Survey (MIS), 40% of children aged 6–59 months tested positive for malaria; parasite prevalence was twice as high in rural compared to urban areas (49% vs. 25%) and was highest in the northern region (52%) [16].

In 2010, the government of Sierra Leone recognized the importance of improving access to essential medications, including anti-malarials, to reduce childhood morbidity and mortality. Two initiatives were rolled out to improve access to health care: (1) The Free Health Care Initiative (FHCI), which provides services and medications free of charge to pregnant women, lactating mothers and children under five at government health facilities along with supportive supply-side interventions, such as improved drugs and medical supply chains; health workforce strengthening; governance; infrastructure for service delivery; communication; monitoring and evaluation; and health financing [17]; and (2) a malaria treatment policy advocating for free malaria testing and treatment with ACT for all malaria cases [18].

These initiatives only addressed health system factors that impact access and targeting of ACT, steps at the beginning of the effectiveness pathway, and did not focus on the quality of care provided [10, 11, 19]. To be truly effective, health workers must follow malaria treatment guidelines, and patients and caregivers must adhere to the prescribed ACT regimens. If these last steps of the pathway, focusing on adherence, are not realized, effective treatment and control of malaria cannot be achieved. Therefore, it is critical to measure and understand factors associated not only with access, but also adherence to ACT.

Although a number of national surveys between 2013 and 2016 documented access to ACT in Sierra Leone, ranging from 77–97% [14, 16, 20, 21], none of these surveys measured treatment adherence nor factors associated with access and adherence to ACT. In 2012, a nationwide malaria knowledge, attitudes, and practices (mKAP) survey was carried out in Sierra Leone, which included not only questions about treatment access but also treatment adherence [22]. To further explore access and adherence to ACT, a secondary analysis of the mKAP dataset was conducted to quantify, and determine factors associated with, access and adherence to ACT in Sierra Leone.

## Methods

### Study design

The mKAP was conducted in 2012 by Catholic Relief Services in partnership with the National Malaria Control Programme (NMCP), supported by Statistics Sierra Leone. The primary objective of the survey was to gather information to inform and update the national malaria communication strategy. Additionally, data from the survey was used to establish a baseline for malaria control activities that were to be subsequently implemented with support from the Global Fund to Fight AIDS, Tuberculosis, and Malaria [22]. This study is a secondary analysis of a subset of data collected from the mKAP survey.

The mKAP survey was a nationally representative two-stage cluster sample survey conducted in all 14 districts of Sierra Leone. Thirty primary sampling units (PSU) per district were randomly selected using probability proportional to size (PPS) based on estimates from the National Census [23]. This resulted in 5880 randomly chosen households (14 households per PSU; 420 households per district). The questionnaire was based on the Roll Back Malaria standardized guidelines for core population-level indicators [24]. All respondents answered questions about household demographics and assets, malaria knowledge and prevention practices, recent pregnancy experiences, and whether the household contained one or more children aged under-five who had a fever in the previous two weeks. Information on fever treatment was collected on up to three such children per household. The mKAP survey was conducted in 2012 before a question about receiving a blood test was universally introduced into national surveys, and therefore did not include a question on whether the child had “*blood taken from his/her finger or heel for testing.”*

### Data collection

Data were collected by trained field staff using Apple iPhones provided by Catholic Relief Services. The devices were programmed using the iFormBuilder mobile platform (Zerion Software, Inc., Herndon, VA, USA) [25]. All electronic data were transferred from the Apple devices into a cloud database regularly while in the field using the local 3G mobile network. Upon completion of the fieldwork, any remaining forms that needed to be transferred were uploaded via wireless internet connections at Statistics Sierra Leone and Catholic Relief Services offices in Freetown.

Paper questionnaires were provided to teams to use only as a backup in case of electronic equipment failure. When necessary, data entered onto paper forms were then entered into an iPhone as soon as it was possible. Backup files of the database were stored on two external servers (iFormBuilder and a specially created Google email account). Additionally, data were stored on the iPhones until completion of the study. For quality control, validation and built-in skip logic were written into the iFormBuilder program.

### Outcome variables and predictors

The objectives of this study were: (1) to quantify the level of access and adherence to ACT in children less than five in Sierra Leone, (2) to assess factors associated with access to ACT for children under-five with fever in the two weeks preceding the survey, and (3) to identify factors associated with adherence to ACT in those children that received ACT for their fever. Access was defined as receiving ACT for the treatment of the most recent fever. Adherence was defined as taking the treatment for the recommended 3 days. The methodology used to assess adherence was similar to that used in two previous cross-sectional household studies in Kenya, which utilized a self-report question to assess whether the duration of treatment with ACT was correct (i.e. 3 days) [26, 27]. Those taking ACT for 3 days were considered to have completed treatment and were classified as adherent, while those taking ACT for less than 3 days or more than 3 days were classified as non-adherent.

**Fig. 1:**
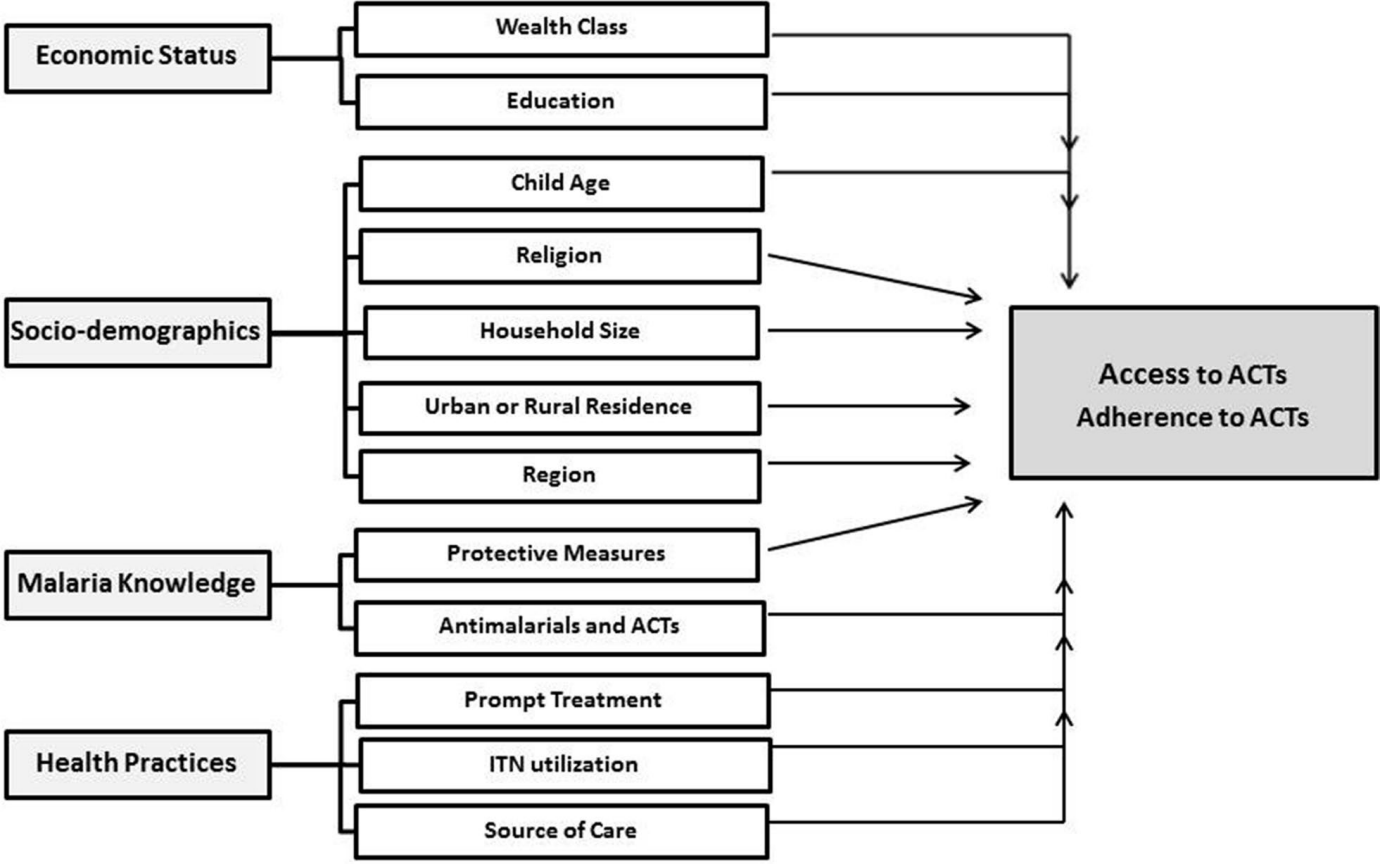
Conceptual framework. A summary of hypothesized socio-demographic factors associated with access and adherence to ACT

Using the available variables from the survey dataset, along with the factors identified in the ACT adherence literature, a list of *a priori* predictors were identified and evolved into a conceptual framework (Fig. 1). Four categories of potential predictors of access and adherence were identified: (1) socioeconomic status (i.e. wealth class and education); socio-demographic characteristics (i.e. child age, religion, household size, place of residence); (2) knowledge of malaria (i.e. knowledge of protective measures and treatments); and (3) health practices (i.e. accesses prompt treatment for fever, ITN utilization, and source of health care).

### Data analysis

Stata Version 12 (StataCorp, College Station, TX USA) and Excel (Microsoft Corp. Redmond, WA, USA) were used for data processing and analysis. In all analyses, the “svy” commands were used to account for the survey design, including clustering by PSU and stratification by the location of the PSU (urban/rural). Descriptive statistics were used to summarize household and respondent characteristics as well as treatment-seeking behaviour for children with fever. Household socioeconomic status was based on a principal component analysis (PCA) of household assets, split into tertiles [28].

Logistic regression models were used to estimate the crude and adjusted odds ratios and their 95% confidence intervals to assess the strength of the association between the *a priori* predictors and the two outcomes (access and adherence to ACT). All predictor variables were included in multivariable analyses regardless of p-values, with the exception that for any pair of covariates identified to be strongly correlated (Pearson’s correlation r ≥ 0.8), one was removed from the final model. Associations between the predictors and outcomes were considered significant if the p-value was < 0.05.

### Ethical considerations

The original study protocol was approved by the Sierra Leone Ethics and Scientific Review Committee prior to the commencement of activities. Ethical approval to conduct this secondary analysis of the mKAP data set was obtained from the London School of Hygiene & Tropical Medicine. Permission to use this data for secondary analysis was obtained from CRS and the NMCP in Sierra Leone.

## Results

### Survey profile

**Fig. 2:**
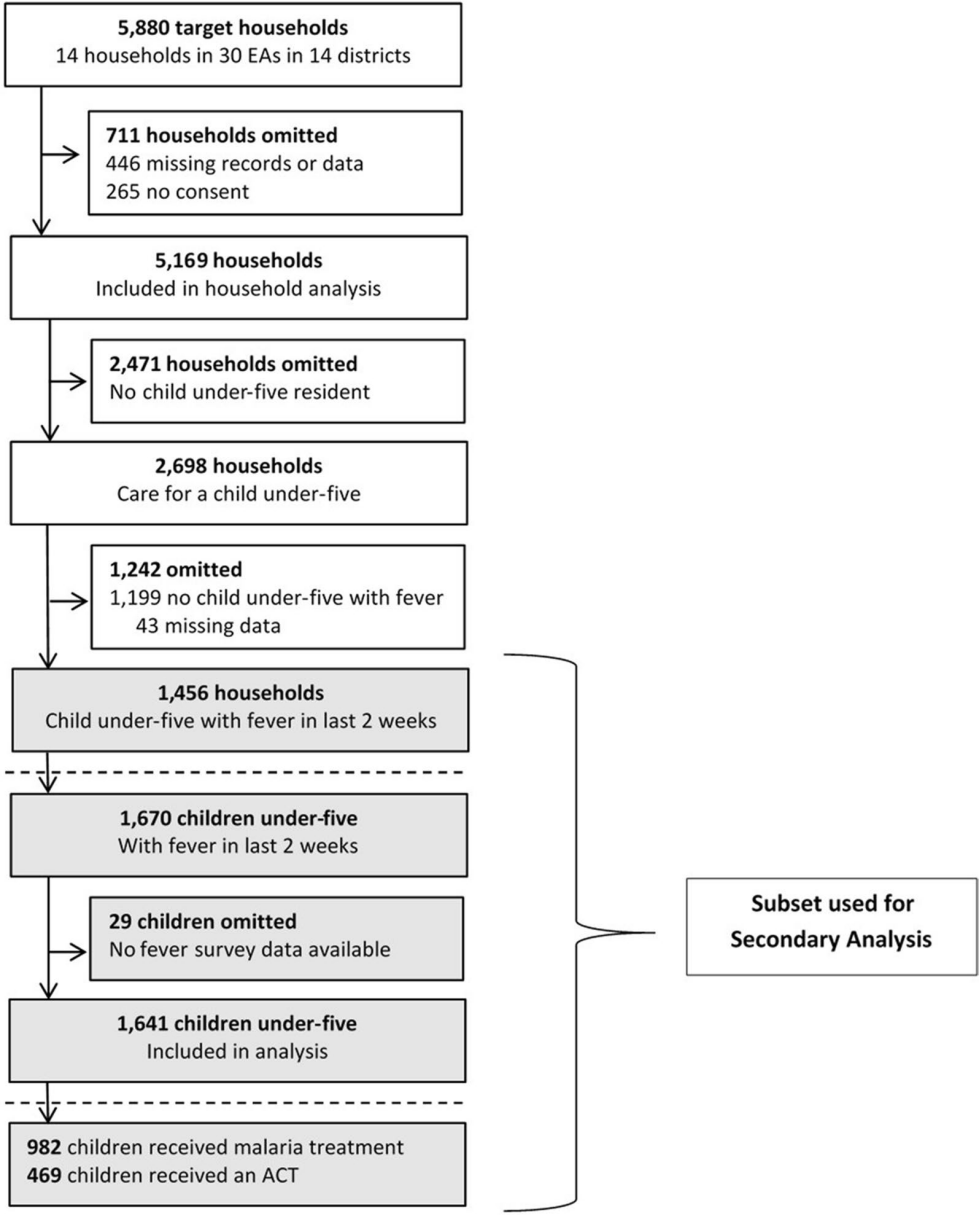
mKAP survey profile

Of 5169 enrolled households, 1456 reported at least one child under-five with fever in the past two weeks and were included in the analysis (Fig. 2). Access to ACT was assessed in 1641 children residing in these households who had a recent history of fever and data on treatment available. Of these children, factors associated with access to ACT were estimated for children whose caregiver sought and received any treatment for their child’s fever (n = 982). Factors associated with adherence to ACT were assessed for children who received ACT and had data on treatment duration (n = 466).

### Characteristics of households and their children

Overall, households that reported fever in a child under-five within the past two weeks were similar to other households, as were the adult respondents from these households (Table 1). Households with febrile children were primarily located in rural areas (87.6%), practiced the Islamic faith (81.0%), and owned at least one bed net of any type (87.2%). The average age of the adult caregiver respondents was 39.2 years, and 66.8% reported not having any formal education. Three out of four adult respondents (76.6%) reported sleeping under an insecticide-treated net (ITN) the previous night. Although most respondents were knowledgeable about malaria, some information gaps were identified, specifically regarding the prevention and treatment of malaria. Only 606 (41.8%) respondents were aware that ACT was the recommended treatment for malaria.

**Table 1:** Household and individual-level characteristics of survey participants, stratified by the presence or absence of a child under five with fever in the preceding 2 weeks

Variable	Categories	Under-five Households with fever (N = 1456)		Under-five Households without fever (N = 1199)	
		n(%)	95% CI	n(%)	95% CI
Household					
Location	Urban	191 (12.4%)	10.6–14.4%	184 (14.8%)	12.8–17.0%
	Rural	1265 (87.6%)	85.6–89.4%	1015 (85.2%)	83.0–87.2%
Region of Sierra Leone	North	672 (46.9%)	41.4–52.5%	368 (30.6%)	25.8–35.9%
	South	371 (25.4%)	21.1–30.3%	427 (35.7%)	30.4–41.4%
	East	291 (19.5%)	15.7–24.1%	236 (19.8%)	15.9–24.5%
	West	122 (8.2%)	5.8–11.3%	168 (13.9%)	10.6–18.0%
Number of household residents	1–5	430 (29.3%%)	26.5–32.2%	440 (36.6%)	33.6–39.8%
	6–10	795 (54.7%)	51.8–57.5%	602 (50.4%)	47.3–53.5%
	11+	231 (16.1%)	13.9–18.5%	157 (12.9%)	11.0–15.2%
Religion	Christian	284 (19.0%)	16.2–22.2%	261 (21.9%)	18.9–25.4%
	Muslim	1172 (81.0%)	77.8–83.8%	938 (78.1%)	74.8–81.2%
Socio-economic status	1 (poorest)	508 (35.4%)	31.9–39.0%	400 (33.4%)	30.0–37.1%
	2	532 (36.2%)	33.3–39.1%	410 (34.2%)	31.2–37.4%
	3 (least poor)	416 (28.5%)	25.2–31.9%	389 (32.3%)	28.6–36.3%
Ownership of bed nets	Own any net	1272 (87.2%)	10.1–15.1%	1045 (86.9%)	84.4–89.1%
	Net is an ITN	1214 (83.1%)	80.5–85.5%	989 (82.2%)	79.2–84.9%
	Mean ITNs	2.6	2.5–2.8	2.5	2.4–2.6
Household adult respondents					
Mean age (years)		39.2	38.4–40.1	38.7	37.8–39.6
Gender	Male	684 (47.1%)	44.0–50.2%	577 (48.2%)	45.0–51.5%
	Female	772 (52.9%)	49.8–56.0%	622 (51.8%)	48.5–55.0%
Education	None	967 (66.8%)	63.9–69.6%	779 (64.7%)	61.4–67.9%
	Primary	173 (11.8%)	10.1–13.8%	124 (10.7%)	8.9–12.7%
	Secondary/higher^a^	279 (18.7%)	16.4–21.2%	277 (23.0%)	20.3–25.9%
	Arabic/Other	37 (2.7%)	1.9–3.8%	19 (1.7%)	1.0–2.8%
ITN use previous night	Respondent	1129 (76.6%)	73.7–79.3%	913 (76.2%)	72.9–79.2%
Knowledge of malaria-related topics					
Ever hear of the illness called “Malaria”	Yes	1492 (98.1%)	97.0–98.8%	1167 (97.4%)	96.1–98.3%
At least one sign or symptom of malaria	Yes	1374 (94.2%)	92.5–95.6%	1116 (93.0%)	91.1–94.6%
All are susceptible to malaria	Yes	1065 (73.2%)	70.3–75.9%	884 (73.4%)	70.3–76.3%
At least one malaria protective measure	Yes	1244 (85.2%)	82.6–87.5%	1026 (85.4%)	82.7–87.8%
ITNs can prevent malaria	Yes	865 (59.6%)	56.3–62.9%	741 (60.9%)	57.1–64.5%
At least one antimalarial drug	Yes	1268 (86.9%)	84.7––88.8%	1021 (85.1%)	82.5–87.3%
Recommended treatment with ACTs	Yes	606 (41.8%)	38.5–45.3%	527 (43.7%)	40.2–47.2%

^a^Secondary or higher includes technical and vocational school

### Treatment for children under‐five with fever

Of the 1641 children under-five with a fever episode in the last 2 weeks, care was sought for 1038 (63.4%) (Table 2). The majority of children (82.2%) were taken to a public health facility. Of those children for whom treatment was sought, most (n = 982) received any treatment, the majority of which were anti-malarials (n = 780). Of these children, 469 (47.6%) received ACT, most of whom (n = 314; 67.8%) were treated within 24 h of onset. Only 222 (47.4%) children with fever received ACT and completed 3-day treatment, and only 137 (31.6%) received prompt treatment (within 24 h) with ACT and completed the 3-day treatment.

**Table 2:** Treatment and treatment-seeking behaviors for children under-five with fever (n = 1641)

	Observations			
	n/N	%	Linearized SE	95% CI
Treatment seeking for fever				
Caregiver sought treatment	1038/1641	63.4 %	1.67%	60.1–66.6%
Caregiver sought prompt treatment (≤ 24 h)	571/1038	55.9%	1.87%	52.2–59.6%
First treatment source^a^				
Public health facility	854/1038	82.2%	1.48%	79.1–84.9%
Other^b^	184/1038	17.8%	1.48%	15.1–20.9%
Treatment of fever				
Received any treatment	982/1038	94.6%	0.83%	92.7–96.1%
Type of treatment				
Artemisinin combination therapy (ACT)	469/982	47.6%	2.11%	43.4–51.7%
Chloroquine	472/982	48.7%	2.19%	44.5–53.1%
Sulfadoxine-pyrimethamine (SP)	153/982	15.0%	1.37%	12.5–17.9%
Paracetamol	770/982	78.4%	1.67%	74.3–80.9%
Herbs	141/982	14.4%	1.36%	11.6–17.0%
Other^c^	136/982	13.5%	1.32%	11.1–16.3%
ACT treatment time^d^				
Received ACT same/next day ( within 24 h)	314/469	67.8%	2.33%	63.0–72.2%
Received ACT on day 2	93/469	19.4%	2.00%	15.8–23.7%
Received ACT 3 + days	29/469	6.0%	1.07%	4.2–8.5%
ACT treatment time unknown	33/469	6.8%	1.21	4.8–9.6%
ACT duration^d^				
Took ACT for 3 days (correct duration)	222/469	47.4%	2.56%	42.4–52.4%
Did not take ACT for 3 days	244/469	52.0%	2.53%	47.1–57.0%
ACT duration unknown	3/469	0.57%	0.03%	0.19–1.74%
Mean duration of ACT treatment (days)		3.62	0.80	3.47–3.78
Prompt and effective treatment with ACT^e^				
Children who received ACT < 24 h & Completed 3-day treatment course	137/433	31.6%	2.57%	27.3–37.4%

^a^The denominator is the number seeking treatment for the fever in the last 2 weeks (n = 1038)

^b^Other sources of treatment include: Community Health workers [Community Health Worker (CHW), Traditional Birth Attendant (TBA), Blue Flag Volunteer (BFV)] = 39; Informal Health workers (drug peddler, traditional healer) = 31; Drug shops/Pharmacy = 96; private clinics/doctors = 9 and self-treatment = 9

^c^Other drugs received include other antimalarial–mono-therapy: quinine and amodiaquine (4), antibiotics (34), antidiarrheal/ORS (17), other antipyretic (7), cough medicine (2), deworm (1), vitamins (12), iron (16), unnamed syrup (6), injection (11), routine medication (10), unspecified/unknown (16)

^d^The denominator is the number who received ACT (n = 469)

^e^The denominator is the number who received ACT and who have information on the timing and duration of treatment (n = 433)

### Factors associated with access

In a multivariate analysis restricted to children who received any treatment for fever (n = 982), five factors were found to be significantly associated with receiving ACT (access), including geographic region, the knowledge that ITNs protect against malaria, knowledge of ACT, older child age, and seeking care at a public health facility (Table 3). ACT access was highest in the eastern region. Knowledge of ACT was the strongest predictor; children with caregivers who were knowledgeable about ACT had almost three times the odds of receiving an artemisinin-based combination for treatment of fever than children whose caregivers lacked this knowledge (OR: 2.78; 95% CI 2.02–3.80; p < 0.001). Similarly, children whose caregiver knew that ITNs provide protection from malaria were more likely to receive ACT (OR: 1.84; 95% CI 1.29–2.63; p = 0.001). Children treated at a public health facility had nearly twice the odds of receiving an artemisinin-based combination compared to those treated elsewhere (OR: 1.86; 95% CI 1.27–2.72; p = 0.001), and older children (24–59 months) were more likely to receive ACT than children aged 0–23 months (OR: 1.45; 95% CI 1.07–1.96; p = 0.016).

**Table 3:** Factors associated with receiving an ACT among febrile children under-five who received any treatment (n = 982)

Variable	n/N	%	Unadjusted analysis		Adjusted analysis	
			OR (95% CI)	p value	OR (95% CI)	p value
Location						
Rural	386/835	46.3%	Ref	0.181	Ref	0.899
Urban	83/147	54.9%	1.41 (0.85–2.33)		1.03 (0.63–1.69)	
Region						
West	36/75	47.8%	Ref	0.253	Ref	0.025
North	203/452	45.0%	0.89 (0.51–1.56)		1.20 (0.64–2.26)	
South	125/268	45.9%	0.93 (0.51–1.70)		1.48 (0.79–2.83)	
East	105/187	56.4%	1.41 (0.75–2.66)		2.30 (1.20–4.44)	
Household size						
1–5	146/269	52.2%	Ref	0.058	Ref	0. 178
6–10	257/548	47.6%	0.83 (0.59–1.17)		0.95 (0.68–1.33)	
11+	66/165	39.9%	0.61 (0.41–0.91)		0.69 (0.46–1.04)	
Household religion						
Muslim	375/796	46.9%	Ref	0.402	Ref	0.579
Christian	94/186	50.7%	1.17 (0.81–1.67)		0.90 (0.62–1.30)	
Socio-economic status						
1 (poorest)	131/317	41.0%	Ref	0.001	Ref	0.237
2	175/382	46.1%	1.23 (0.87–1.73)		1.00 (0.68–1.47)	
3 (least poor)	163/283	57.0%	1.91 (1.31–2.77)		1.41 (0.88–2.24)	
Respondent education						
None	290/644	45.1%	Ref	0.059	Ref	0.930
Primary	63/123	51.2%	1.28 (0.87–1.89)		1.03 (0.68–1.58)	
Secondary or higher	107/196	53.8%	1.42 (0.99–2.03)		1.00 ( 0.66–1.50)	
Arabic school or Other	9/19	47.9%	1.12 (0.44–2.87)		1.41 (0.49–4.08)	
Everyone is at risk						
No	120/250	47.4%	Ref	0.948	Ref	0.380
Yes	349/732	47.6	1.01 (0.73–1.39)		0.85 (0.60–1.22)	
ITNs protect from malaria						
No	129/370	35.0%	Ref	< 0.001	Ref	0.001
Yes	340/612	55.2%	2.29 (1.66–3.15)		1.84 (1.29–2.63)	
Under 5 slept under ITN						
No	119/287	40.2%	Ref	0.009	Ref	0.061
Yes	350/695	50.6%	1.53 (1.11–2.10)		1.41 (0.98–2.02)	
Knowledge of ACTs						
No	187/537	34.7%	Ref	< 0.001	Ref	< 0.001
Yes	282/445	63.0%	3.20 (2.36–4.35)		2.78 (2.02–3.80)	
Child age (months)						
0–23 months	136/321	42.6%	Ref	0.047	Ref	0.016
24–59 months	333/661	49.9%	1.34 (1.00–1.79)		1.45 (1.07–1.96)	
Prompt treatment (< 24 h)						
No	209/440	47.2%	Ref	0.842	Ref	0.866
Yes	260/542	47.9%	1.03 (0.77–1.37)		1.03 (0.76–1.38)	
Public health facility						
No	57/159	36.4%	Ref	0.004	Ref	0.001
Yes	412/823	49.7%	1.73 (1.19–2.52)		1.86 (1.27–2.72)	

### Factors associated with adherence

In a multivariate analysis restricted to children who received ACT and had data on treatment duration (n = 466), only one factor was found to be significantly associated with adherence (Table 4). Children receiving ACT within 24 h of symptom onset were less likely to complete treatment than those who received ACT treatment beyond this window (OR: 0.55; 95%CI 0.34–0.89; p = 0.015). Due to collinearity with ‘knowledge of ACT,’ ‘knowledge of at least one anti-malarial’ and ‘knowledge of at least one sign or symptom of malaria’ were removed from the multivariable models. Similarly, ITN use was retained, while ITN ownership and knowledge of any malaria protective measures were removed. As knowledge of the term ‘malaria’ was collinear with both knowledge of ACT and ITN utilization, it was removed from both final multivariable models.

**Table 4:** Factors associated with adherence to ACTs among febrile children under-five who received treatment with an artemisinin-based combination (n = 466)

Variable	n/N	%	Unadjusted analysis		Adjusted analysis	
			OR (95% CI)	p value	OR (95% CI)	p value
Location						
Rural	178/385	46.2%	Ref	0.233	Ref	0.245
Urban	44/81	55.3%	1.44 (0.79–2.64)		1.49 (0.76–2.95)	
Region						
West	11/35	30.1%	Ref	0.355	Ref	0.211
North	98/202	47.9%	2.13 (0.83–5.50)		2.58 (0.99–6.77)	
South	60/124	49.3%	2.26 (0.87–5.89)		2.83 (1.04–7.68)	
East	53/105	50.8%	2.40 (0.91–6.32)		2.76 (1.04–7.32)	
Household size						
1–5	63/144	45.3%	Ref	0.487	Ref	0.620
6–10	122/256	47.3%	1.08 (0.72–1.62)		1.13 (0.73–1.73)	
11 +	37/66	54.2%	1.43 (0.79–2.59)		1.35 (0.73–2.50)	
Household religion						
Muslim	172/373	46.0%	Ref	0.158	Ref	0.058
Christian	50/93	54.7%	1.42 (0.87–2.30)		1.61 (0.98–2.65)	
Socio-economic status						
1 (poorest)	62/131	46.5%	Ref	0.883	Ref	0.930
2	83/175	47.0%	1.02 (0.64–1.63)		1.07 (0.66–1.75)	
3 (least poor)	77/160	49.4%	1.13 (0.68–1.86)		1.12 (0.60–2.08)	
Respondent education						
None	138/288	48.1%	Ref	0.984	Ref	0.854
Primary	30/63	48.5%	1.02 (0.58–1.80)		0.87 (0.46–1.64)	
Secondary or higher	50/106	46.5%	0.94 (0.57–1.54)		0.80 (0.46–1.38)	
Arabic or other	4/9	42.3%	0.79 (0.18–3.49)		0.74 (0.17–3.26)	
Know everyone is at risk						
No	60/120	51.3%	Ref	0.390	Ref	0.329
Yes	162/346	46.4%	0.82 (0.53–1.29)		0.79 (0.49–1.27)	
Know ITNs protect						
No	62/128	48.4%	Ref	0.844	Ref	0.943
Yes	160/338	47.4%	0.96 (0.63–1.47)		0.98 (0.63–1.54)	
U5 slept under ITN						
No	58/118	48.8%	Ref	0.779	Ref	0.829
Yes	164/348	47.3%	0.94 (0.61–1.44)		0.95 (0.60–1.50)	
Knowledge ACTs						
No	88/187	46.9%	Ref	0.805	Ref	0.866
Yes	134/279	48.2%	1.05 (0.70–1.57)		1.04 (0.67–1.60)	
Child age (months)						
0–23 months	60/135	43.9%	Ref	0.276	Ref	0.270
24–59 months	162/331	49.2%	1.24 (0.84–1.81)		1.26 (0.83–1.90)	
Prompt treatment (< 24 h)						
No	102/207	49.5%	Ref	0.744	Ref	0.150
Yes	119/258	45.9%	1.05 (0.79–1.39)		1.25 (0.92–1.70)	
Public health facility						
No	28/57	48.3%	Ref	0.926	Ref	0.951
Yes	194/409	47.6%	0.97 (0.55–1.74)		0.98 (0.53–1.81)	
ACT within 24 h						
No	85/154	55.0%	Ref	0.047	Ref	0.015
Yes	137/312	44.1%	0.65 (0.42–0.99)		0.55 (0.34–0.89)	

Data for ACT duration was available for 466 children out of the 469 that received ACT

## Discussion

The pathway to effective malaria case management depends on timely access to ACT and patient (or caregiver) adherence to treatment regimens. ACT has been recommended as the first-line treatment in Sierra Leone since 2004; however, in 2012, uptake remained suboptimal. To further investigate access to ACT and adherence to treatment guidelines in Sierra Leone, a secondary analysis of the national 2012 mKAP survey was undertaken. In a previous nationwide survey conducted in 2010, only 19.2% of febrile children received ACT, and only 50.3% received any anti-malarial within 24 h [14]. Assuming that a large proportion of febrile illness in children under five in Sierra Leone is due to malaria, these results suggest that challenges to delivering prompt and effective malaria treatment still remained in 2012.

In 2012, most children under-five with fever in the 2 weeks before the survey did not receive an artemisinin-based combination. ACT access varied geographically and was highest in the eastern region of the country. Similar to findings reported from Thailand, Kenya, and Uganda [29–31], this study found that children were more likely to receive ACT if their caregiver had prior knowledge of ACT and ITNs, suggesting that health education interventions could improve patient access to ACT. Additionally, this analysis found that seeking care from a public health facility doubled the odds of receiving ACT, suggesting that price or limited availability may impact access to ACT in the private sector. This is similar to other studies in sub-Saharan Africa, which have reported improved access to ACT for children treated in the public sector [32, 33].

This study also demonstrates a difference in access based on age, with older children (2–4 years) more likely to receive ACT than younger children (< 2 years). While removing user-fees removes the cost barrier of accessing care for individuals, it can strain weak health systems when demand increases. Higher patient loads require more resources to provide adequate service delivery. Although Sierra Leone had a relatively high availability of amodiaquine + artesunate (the ACT of choice at the time) compared to other post-conflict countries [34], the number of infant doses has often been insufficient due to improper forecasting and provision of pediatric formulations [35, 36]. Such challenges could be even more significant with the recent Ebola outbreak and ongoing pandemic [7, 8].

Of those febrile children who received artemisinin-based combination, less than half completed the recommended 3-day course of treatment (47.6%). However, no significant association between knowledge of ACT, malaria, or prevention practices and adherence was found, despite suggestions that patient knowledge, attitudes, and beliefs may be strong predictors of adherence [15, 31, 37–40]. Although Bruxvoort et al. reported that age, higher household income, higher education level, malaria knowledge, and treatment-seeking behavior are factors facilitating anti-malarial adherence [41], none of the *a priori* socioeconomic or demographic factors were associated with adherence in this study.

Unlike the findings reported for access, this analysis found no association between the source of care and treatment adherence. Unexpectedly, poor adherence to ACT was associated with accessing ACT promptly (within 24 h from the onset of symptoms). Lemma et al.. reported similar results; participants that delayed 1 day before seeking treatment were more adherent than those seeking prompt treatment (OR: 5.39; 95% CI 1.83–15.88) [37]. In contrast, a study in Uganda reported that prompt access to ACT was associated with higher treatment adherence [30]. Similarly, patients in Kenya seeking treatment greater than 1 day after the start of fever were 27% less likely to be adherent [31]. Given these mixed results, the association between prompt treatment for fever and lower ACT adherence should be interpreted with caution. Those accessing treatment early may have had lower parasite loads, which was cleared more quickly, resulting in fewer symptoms and possibly lower treatment adherence. Moreover, as the mKAP survey did not capture information on confirmatory malaria diagnosis, the child may have had a non-malaria febrile illness, and their symptoms may have resolved at the same time as receiving ACT, thus leading the caregiver to discontinue the treatment.

National cross-sectional household surveys such as the Demographic Health Survey (DHS), Multiple Indicator Cluster Survey (MICS), and the Malaria Indicator Survey (MIS) routinely collect information on the treatment of fever. These surveys including questions on treatment-seeking behavior for fever, medications received for that fever, and how soon after the onset of symptoms, the treatment was initiated. Additionally, since 2013, most surveys have gathered information on whether a blood test was received. However, the test question is not malaria-specific, nor is information on the test result collected due to concerns about the reliability of the data [42, 43]. Without this vital specific information, caution should be taken when interpreting results on fever case management from these surveys, as these data would represent the treatment of fever and not necessarily malaria. Ashton et al.. recently found that caregiver recall surrounding testing and diagnosis to be valid; therefore, the recommendation regarding confirmed malaria cases may need to be reviewed [44].

The fever case management sections of these national surveys could be expanded to include questions related to ACT treatment adherence, such as duration and/or completion of treatment, as was done in this study. Including these additional questions would allow a population-level estimate of adherence to be measured, as well as information on access to ACT, providing a more complete picture of the malaria treatment pathway. Collecting ACT adherence data through national surveys would be simple to implement, sustainable, and cost-effective. However, the use of national surveys to assess ACT adherence has several limitations: (1) the method assumes that respondents know and recognize which anti-malarial or artemisinin-based combination was prescribed for their child; (2) the data is relying on self-reported outcomes; and (3) unless surveys collect information about diagnostic testing specifically for malaria along with those test results, then the utility of adherence data would be limited as it would apply only to children with fever who may or may not have malaria.

Although this was a national survey, which allows generalizability of the findings to the entire country, there were some limitations. First, the data presented here were collected in 2012. Despite the delay in reporting these results, ACT adherence at the national level remains unknown in 2020, and factors impacting access and adherence to ACT have yet to be evaluated in Sierra Leone. Second, the study cannot provide a causal relationship between improved access and government programs rolled out to improve access to health services. However, the successful implementation of the FHCI in certain districts may have contributed to better access to medicines and services [19]. Third, the analysis was limited to the variables collected and may not have captured all the factors plausibly associated with receiving or completing treatment with ACT. In particular, this secondary analysis did not contain information on ACT and malaria rapid diagnostic tests stock-outs, confirmatory diagnosis of malaria, the quality of care at the health facility (including if the health worker provided information on how to administer the medication), or treatment completion. Additionally, questions on the number of tablets taken or whether treatment was completed were not included in this survey and would have contributed to a more precise quantification of adherence. Finally, although the sample size was large, it may not have provided the optimal power needed to detect associations between adherence and the *a priori* socioeconomic or demographic factors identified.

## Conclusions

This study demonstrates that poor access and adherence to ACT remained key challenges to ensuring effective malaria case management in Sierra Leone in 2012 and continues to be a challenge in the face of public health emergencies such as Ebola and COVID-19. While efforts have been made to improve access to key health services in Sierra Leone, such as malaria treatment, further emphasis on ACT adherence is still needed. Optimizing the supply chain, implementation of the Free Health Care Initiative, and scaling up malaria communication campaigns to include messages on adherence could improve malaria treatment effectiveness in Sierra Leone. Finally, national household surveys could be expanded to capture key indicators on ACT access and adherence to help guide malaria case management in the future.

## Data Availability

The data used for this study were made available by Catholic Relief Services, specifically for this analysis. Data are available from the authors upon reasonable request and with permission of Catholic Relief Services.

## Abbreviations

ACT: Artemisinin-based combination therapy
AL: Artemether–lumefantrine
AOR: Adjusted odds ratio
AQAS: Fixed-dose combination amodiaquine–artesunate
AQ+AS: Co-packaged amodiaquine plus artesunate
CRS: Catholic Relief Services
DHS: Demographic Health Survey
FHCI: Free Health Care Initiative
GFATM: The Global Fund to Fight AIDS, Tuberculosis, and Malaria
ITN: Insecticide-treated net
KAP: Knowledge, attitudes, and practices
MIS: Malaria Indicator Survey
MICS: Multiple Indicator Cluster Survey
mKAP: Malaria Knowledge, Attitudes, and Practices Survey
NMCP: National Malaria Control Programme
OR: Odds ratio
PCA: Principal component analysis
PPS: Probability proportional to size
PSU: Primary sampling units
WHO: World Health Organization
95% CI: 95% confidence interval
